# Validation of extravascular lung water measurement by single transpulmonary thermodilution: human autopsy study

**DOI:** 10.1186/cc9250

**Published:** 2010-09-06

**Authors:** Takashi Tagami, Shigeki Kushimoto, Yasuhiro Yamamoto, Takahiro Atsumi, Ryoichi Tosa, Kiyoshi Matsuda, Renpei Oyama, Takanori Kawaguchi, Tomohiko Masuno, Hisao Hirama, Hiroyuki Yokota

**Affiliations:** 1 Department of Emergency and Critical Care Medicine, Aidu Chuo Hospital, 1-1 Tsuruga, Aiduwakamatsu, Fukushima, 965-8611, Japan; 2 Department of Emergency and Critical Care Medicine, Nippon Medical School, 1-1-5 Sendagi, Bunkyo-ku, Tokyo, 113-8613, Japan; 3 Tokyo Rinkai Hospital, 1-4-2 Rinkaicho, Edogawa-ku, Tokyo, 134-0086, Japan; 4 Department of Emergency and Critical Care Medicine, Yamanashi Central Hospital, 1-1-1 Fujimi, Kofu, Yamanashi, 400-8506, Japan; 5 Department of Surgery, Saiseikai Chuo Hospital, 1-4-17 Mita, Minato-ku, Tokyo, 108-0073, Japan; 6 Department of Pathology, Aidu Chuo Hospital, 1-1 Tsuruga, Aiduwakamatsu, Fukushima, 965-8611, Japan

## Abstract

**Introduction:**

Gravimetric validation of single-indicator extravascular lung water (EVLW) and normal EVLW values has not been well studied in humans thus far. The aims of this study were (1) to validate the accuracy of EVLW measurement by single transpulmonary thermodilution with postmortem lung weight measurement in humans and (2) to define the statistically normal EVLW values.

**Methods:**

We evaluated the correlation between pre-mortem EVLW value by single transpulmonary thermodilution and post-mortem lung weight from 30 consecutive autopsies completed within 48 hours following the final thermodilution measurement. A linear regression equation for the correlation was calculated. In order to clarify the normal lung weight value by statistical analysis, we conducted a literature search and obtained the normal reference ranges for post-mortem lung weight. These values were substituted into the equation for the correlation between EVLW and lung weight to estimate the normal EVLW values.

**Results:**

EVLW determined using transpulmonary single thermodilution correlated closely with post-mortem lung weight (*r *= 0.904, *P *< 0.001). A linear regression equation was calculated: EVLW (mL) = 0.56 × lung weight (g) - 58.0. The normal EVLW values indexed by predicted body weight were approximately 7.4 ± 3.3 mL/kg (7.5 ± 3.3 mL/kg for males and 7.3 ± 3.3 mL/kg for females).

**Conclusions:**

A definite correlation exists between EVLW measured by the single-indicator transpulmonary thermodilution technique and post-mortem lung weight in humans. The normal EVLW value is approximately 7.4 ± 3.3 mL/kg.

**Trial registration:**

UMIN000002780.

## Introduction

Pulmonary edema is one of the most common problems in critically ill patients and has a profound effect on patient outcome [1,2]. In general, pulmonary edema is diagnosed on the basis of patient history, physical examination, routine laboratory examination, and chest radiographic findings [2,3]. However, interpretation of these parameters is often limited by a certain degree of subjectivity that may cause interobserver error even among experts [4,5]. In addition, clinical symptoms may be undetectable in the incipient stages of edema. The difficulties faced during quantification of pulmonary edema were addressed many years ago [6-8]. However, attempts to develop direct or indirect methods of measuring edema turned out to be lacking in either sensitivity or specificity.

The introduction of the double-indicator thermodilution technique made it possible to measure extravascular lung water (EVLW) and demonstrated excellent correlation between *in vivo *and postmortem gravimetric EVLW values in both animal and human lungs [9,10]. However, this method was cumbersome and too technically challenging for application in routine clinical practice. Therefore, it remained largely a research tool.

For EVLW evaluation in the clinical setting, the double-indicator technique has been replaced by the single-indicator technique, which is implemented in the PiCCO monitoring system (Pulsion Medical Systems, Munich, Germany). EVLW measured by this method has been shown to correlate closely with both the double-indicator technique [11,12] and the gravimetric measurement of lung weight in experimental animal models [13-15]. However, the correlation between single-indicator EVLW and postmortem lung weight in humans has not yet been studied.

Furthermore, validated normal EVLW values by both the double- and single-indicator methods remain unreported. In general, the standard method for determining a normal value is to define and obtain a healthy population of at least 120 individuals [16]. In 1983, Sibbald and colleagues [17] defined the normal mean EVLW as 5.6 mL/kg (3.0 to 8.8 mL/kg) by using the double-indicator technique. However, they included only 16 patients and all of the 'normal' patients were critically ill and mechanically ventilated without pulmonary edema diagnosed on the basis of portable chest roentgenogram findings. A similar definition was reported in 1986 by Baudendistel and colleagues [18], who used the single-indicator method and reported that a mean EVLW of 5.1 mL/kg (2.4 to 10.1 mL/kg) obtained from 6 'normal' critically ill patients constituted the 'normal' EVLW content in the human lung. These 'normal' critically ill patients remained free of both radiographic abnormalities typical of pulmonary edema and physiological evidence of pulmonary dysfunction. However, several studies have indicated that in critically ill patients, chest roentgenograms are not accurate for monitoring modest changes in lung water and that gas exchange abnormalities or dyspnea appears only when EVLW reaches twice its baseline level [6,19].

So far, no study has defined normal EVLW values using the PiCCO system. Most clinical studies have been conducted on critically ill patients as subjects who would not present with normal EVLW [11,20]. In several clinical studies, researchers have considered EVLW values of below 7 mL/kg to be normal [21-26]. However, others have reported EVLW values of below 10 mL/kg to be normal [27-29]. Recently, Craig and colleagues [21] argued that there is a lack of consensus as to what constituted a normal value. Therefore, our study aimed (a) to validate EVLW accuracy using the PiCCO system by postmortem lung weight measurement of the human lung and (b) to define normal EVLW values.

## Materials and methods

This study was approved by our institutional review board and was registered with the University Hospital Medical Information Network Clinical Trials Registry (UMIN-CTR ID UMIN000002780). The study involved the following three processes.

### 1. Examination of the correlation between single-indicator EVLW and postmortem lung weight

We studied 30 consecutive autopsy cases (24 males and 6 females) in which EVLW was measured using the PiCCO system just prior to death from July 2004 to September 2009 in four teaching hospitals. Clinical data were obtained from medical records.

A 4 F or 5 F femoral arterial thermistor-tipped catheter (PV2014L16 or PV2015L20; Pulsion Medical Systems) was inserted in all patients and connected to the PiCCO monitor. The PiCCO monitor uses a single-thermal indicator technique to calculate the cardiac output (CO), global end-diastolic volume (GEDV), EVLW, and other volumetric parameters. A 15-mL bolus of 5% glucose at 5°C was injected through a central venous catheter, and CO was calculated using the Stewart-Hamilton method. Concurrently, the mean transit time and the exponential downslope time of the transpulmonary thermodilution curve were calculated. The product of CO and mean transit time represents the intrathoracic thermal volume (ITTV) [11]. The product of CO and exponential downslope time is the pulmonary thermal volume (PTV) [30]. GEDV is calculated as the difference between the ITTV and PTV, which represents the combined end-diastolic volumes of four cardiac chambers. This allows the calculation of intrathoracic blood volume (ITBV) from the linear relationship with GEDV: ITBV = [1.25 × GEDV] - 28.4 [11]. EVLW is the difference between the ITTV and the ITBV [11,12]. The detailed principles and calculations involved in deriving EVLW using thermodilution techniques are discussed elsewhere [20,31].

The median EVLW value after three bolus injections of 15 mL each was analyzed for each measurement. The absolute EVLW value was indexed to actual body weight (EVLW_a_) and predicted body weight (EVLW_p_), which was calculated as 50 + 0.91 (height in centimeters - 152.4) for males and 45.5 + 0.91 (height in centimeters - 152.5) for females [21,32,33].

To calculate arterial partial pressure of oxygen/fraction of inspired oxygen (PaO_2_/FiO_2 _or P/F) ratio, blood samples were taken via the arterial catheter within 60 minutes before or after the EVLW measurement. Chest roentgenograms were obtained at the bedside on the same day. The correlation between lung injury score (LIS) and EVLW was evaluated to investigate the correlation between EVLW and lung damage. The timing of the EVLW measurement and measurement of other parameters was left to the doctors in charge.

Following death, written informed consent was obtained from the family of each patient prior to autopsy. Experienced pathologists blinded to the study objectives completed all autopsies within 48 hours after the final thermodilution EVLW measurement had been performed by the attending physicians. We chose 48 hours as a cutoff point for inclusion in the study because postmortem lung weight shows little change in the early postmortem period (4.5 to 72 hours) [34]. Prior to autopsy, cadavers were kept in accordance with the policy of each institution. As a result, 23 out of 30 cadavers had been kept in a refrigeration chamber. The remaining 7 cadavers, which had not been refrigerated, underwent autopsy within the 6 hours subsequent to the final EVLW recording.

Body weights and heights of all patients, with the exception of 9 patients whose measurements were performed at the bedside, were measured at autopsy. During autopsy, the weight of both lungs was measured after determining the amount of pleural effusion before formalin fixation.

We derived a linear regression equation after evaluating the correlation between the final EVLW measured by the PiCCO system and postmortem lung weight. We also evaluated the influence of sex, high LIS (>2.5), large volumes of pleural effusion (>500 mL), low cardiac index (CI) (<2.5 L/min per m^2^), high central venous pressure (CVP) (>12 mm Hg), high positive end-expiratory pressure (PEEP) (>10 cm H_2_O), time delay before the autopsy (>24 hours), cause of death as diagnosed by the pathologist (respiratory cause of death or non-respiratory cause of death), and performance of cardiopulmonary resuscitation (CPR) on thermodilution measurements.

### 2. Identification of reference ranges for normal lung weight

The normal value of a clinical measurement is usually defined by Gaussian distribution, which constitutes from the central 95% (or 2 standard deviations [SDs]) value of the healthy population [16,35]. We referred to data from several publications to estimate the normal reference range of human lung weight [36-39]. Sawabe and colleagues [38] reported standard organ weights using data from 1,615 older Japanese patients who died in hospitals in Japan. The age distribution of our study population matched that of the population in their study. Sawabe and colleagues strictly excluded patients with abnormal lungs such as those with pneumonia or diffuse alveolar damage and patients with malignant tumors identified at autopsy. Along with primary exclusions, they excluded organs with off-limit values beyond 99% of bilateral limits. We believe that these criteria make their study protocol particularly robust. Therefore, we considered their data to be representative of normal lung weights.

### 3. Calculation of normal EVLW and EVLW_p _values

Using the linear regression equation for the correlation between transpulmonary EVLW measurement and postmortem lung weight in equation 1 (see Results), we calculated thermodilution EVLW values for normal lungs using the lung weight values reported in the literature. Traditionally, EVLW has been indexed to actual body weight, with the value being expressed as EVLW in milliliters per kilogram. However, several recent clinical studies have found that indexing EVLW to predicted body weight (EVLW_p_), instead of actual body weight (EVLW_a_), improves the predictive value of EVLW for patient survival and correlation with markers of disease severity [21,29,33]. Therefore, we expressed normal EVLW values as EVLW_p_.

### Statistical analysis

Data were presented as mean values ± SD or as the median (interquartile range, IQR), depending on the distribution normality of the variable. In keeping with the literature, reference ranges for lung weights were expressed as mean ± SD. Cadavers were categorized into several groups and were compared using two-sample *t *tests or the Mann-Whitney *U *test for normally and non-normally distributed data, respectively. Postmortem lung weight was compared with EVLW, which was calculated using the single-indicator transpulmonary thermodilution method by Spearman's correlation coefficient (*r*). Because our present study compared the indicator dilution of EVLW (in milliliters) and postmortem lung weight (in grams), we did not use the Bland-Altman plot analysis. It is not possible to analyze different parameters by a Bland-Altman plot analysis. Therefore, we expressed the data in terms of correlation coefficients. The regression line was calculated using Passing and Bablok regression. The difference between any two correlation coefficients was tested by the z test after Gaussian transformation of the coefficients. Reproducibility of EVLW measurements was assessed by the coefficient of variation (CV) and intraclass correlation coefficient (ICC). ICC uses components of variance from a variance analysis and assesses the agreement of quantitative measurements in terms of consistency and conformity [40,41]. The ICC ranges from 0 to 1, where 1 demonstrates perfect reliability. To assess the intraobserver reliability, ICC (1, 1) was used for single-measure reliability and ICC (1, 3) was used for reliability over an average of three measurements. A *P *value of less than 0.05 was considered significant. Statistical analyses were performed using SPSS 17.0 for Windows (SPSS, Inc., Chicago, IL, USA) for all tests except Passing and Bablok regression analysis and comparison of correlation coefficients, which were performed using the software StatFlex 6.0 for Windows (Artech Co. Ltd, Osaka, Japan).

## Results

All autopsies were completed within 48 hours (range of 1 to 47 hours) following the final thermodilution EVLW measurement. Median time from the final measurement to death was 5 hours and 7 minutes. Median time from death to the beginning of the autopsy was 9 hours and 16 minutes, and the median time from the final measurement to the beginning of the autopsy was 17 hours and 39 minutes.

Table 1 summarizes the clinical and autopsy findings. The amount of pleural effusion measured ranged from 10 to 1,600 mL. Twenty-eight patients (93%) were mechanically ventilated and the median PEEP in these patients was 8 cm H_2_O (IQR = 5.0 to 10.0 cm H_2_O). Causes of death diagnosed by a pathologist included the following: multiple organ failure (*n *= 12 patients), pneumonia (*n *= 6), heart failure (*n *= 6), acute respiratory distress syndrome (ARDS) due to sepsis (*n *= 4), and multiple trauma (*n *= 2). Overall, there were 10 patients with respiratory causes of death (RF): 6 patients with pneumonia and 4 patients with ARDS. There were 20 patients without respiratory causes of death (non-RF). The EVLW_p _was significantly higher in the RF group than in the non-RF group (17.1 mL/kg [IQR = 12.9 to 22.0 mL/kg] versus 10.1 mL/kg [IQR = 8.9 to 12.2 mL/kg]; *P *= 0.01). Comparisons of other parameters between RF and non-RF were as follows: lung weight (1,610 g [IQR = 1,500 to 2,120 g] versus 1,212 g [IQR = 960 to 1,360 g]; *P *= 0.004), PaO_2_/FiO_2 _(84.8 ± 49 mm Hg versus 176.0 ± 116 mm Hg; *P *= 0.008), LIS (3 [IQR = 2.3 to 3.6] versus 2 [IQR = 1 to 2.3]; *P *= 0.003), PEEP (8 cm H_2_O [IQR = 6 to 10 cm H_2_O] versus 5 cm H_2_O [IQR = 4 to 9 cm H_2_O]; *P *= 0.17), and pleural effusion (550 mL [IQR = 370 to 850 mL] versus 500 mL [IQR = 300 to 865 mL]; *P *= 0.22).

**Table 1:** Patient characteristics

Characteristics	Value
Age, years	68.0 (60.0-77.0)
Height, m	1.63 (1.56-1.72)
Actual weight, kg	65.0 (54.6-70.0)
Predicted body weight, kg	57.3 (52.4-61.5)
Postmortem lung weight, g	1,320 (1,170-1,620)
Pleural effusion, mL	500 (300-850)
EVLW, mL	655 (553-856)
EVLW_a_, mL/kg	12.0 (8.4-14.4)
EVLW_p_, mL/kg	11.6 (9.7-16.3)
Lung injury score	2.3 (1.3-3.0)
PaO_2_/FiO_2_, mm Hg	145 ± 107
Cardiac index, L/min per m^2^	3.3 ± 1.3

All values are expressed as median (first to third quartile) or as mean ± standard deviation. EVLW, extravascular lung water; EVLW_a_, extravascular lung water indexed to actual body weight; EVLW_p_, extravascular lung water indexed to predicted body weight; PaO_2_/FiO_2_, arterial partial pressure of oxygen/fraction of inspired oxygen.

No difference in lung weight was demonstrated between patients whose autopsy was started within 24 hours (early group; *n *= 20, 1,315 g [IQR = 1,270 to 1,600 g]) and those whose autopsy was started later than 24 hours (late group; *n *= 10, 1,320 g [IQR = 930 to 1,757 g]) (*P *= 0.79).

CPR was performed in 16 cases (53%). Median lung weights were 1,285 g (IQR = 950 to 1,672 g) in the CPR group and 1,430 g (IQR = 1,200 to 1,620 g) in the non-CPR group. There was no statistical difference between the groups (*P = *0.59).

### Reproducibility of EVLW measurements

The CV of EVLW measurement in the present study was 7.4%. ICC (1, 1) and ICC (1, 3) of EVLW measurement in the present study were 0.97 and 0.99, respectively.

### Correlation between single-indicator EVLW and postmortem lung weight

**Figure 1:**
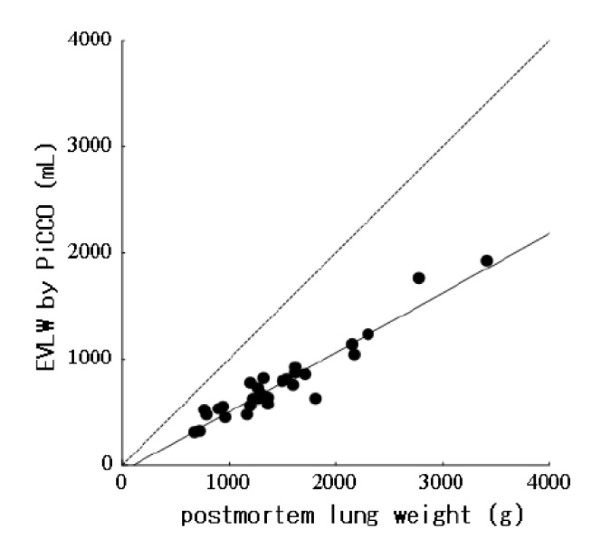
**Correlation of extravascular lung water (EVLW) measured by single transpulmonary thermodilution and by postmortem lung weight**. EVLW (in milliliters) = [0.56 × lung weight (in grams)] - 58.0. *n *= 30, *r *= 0.90, *P *< 0.001. Line of identity is dashed.

We found a very close correlation between transpulmonary measurement of EVLW and postmortem lung weight (*r *= 0.904; *P *< 0.001) (Figure 1). The linear regression equation for correlation was as follows:

(1) EVLW (in milliliters)=[0.56×lung weight (in grams)]−58.0.

For the correlation between transpulmonary measurement of EVLW and postmortem lung weight, no significant difference was observed between sexes (males: *n *= 24, *r *= 0.846, *P *< 0.001; females: *n *= 6, *r *= 0.943, *P *= 0.005; difference of correlation coefficient: *P *= 0.72). Furthermore, no significant difference was found between patients whose pleural effusion amounts were less than or more than 500 mL (≤500 mL: *n *= 13, *r *= 0.89, *P *< 0.001; >500 mL: *n *= 17, *r *= 0.92, *P *< 0.001; difference of correlation coefficient: *P *= 0.13); between low- and high-LIS patients (LIS ≤2.5: *n *= 18, *r *= 0.84, *P *< 0.001; LIS >2.5: *n *= 12, *r *= 0.95, *P *< 0.001; difference of correlation coefficient: *P *= 0.27); or between high- and low-CI patients (CI >2.5 L/min per m^2^: *n *= 20, *r *= 0.84, *P *< 0.01; CI ≤2.5 L/min per m^2^: *n *= 10, *r *= 0.96, *P *< 0.01; difference of coefficient of correlation: *P *= 0.65). Very close correlations were demonstrated with both the high-CVP group (>12 mm Hg; *n *= 13, *r *= 0.94, *P *< 0.01) and the low-CVP group (≤12 mm Hg; *n *= 17, *r *= 0.89, *P *< 0.01), with no statistical difference in coefficient of correlation (*P *= 0.12). Very close correlation was also demonstrated between the high-PEEP group (>10 cm H_2_O; *n *= 9, *r *= 0.95, *P *< 0.01) and the low-PEEP group (≤10 cm H_2_O; *n *= 21, *r *= 0.87, *P *< 0.01), with no statistical difference in the coefficient of correlation (*P *= 0.60). No significant difference was observed between the RF and non-RF groups (RF: *r *= 0.84, *P *< 0.01; non-RF: *r *= 0.93, *P *< 0.01; difference of coefficient of correlation: *P *= 0.39), between the early and late autopsy groups (early versus late: *r *= 0.93, *P *< 0.01 versus *r *= 0.83, *P *< 0.01; difference of coefficient of correlation: *P *= 0.39), or between the groups in which CPR was or was not performed (CPR group: *r *= 0.88, *P *< 0.01; non-CPR group: *r *= 0.90, *P *< 0.01; difference of coefficient of correlation: *P *= 0.68).

### Correlation between single-indicator EVLW and other parameters

A moderate correlation was found between LIS and lung weight/predicted body weight (PBW) (*r *= 0.56, *P *< 0.001). A similar result was found between LIS, EVLW_p _(*r *= 0.61, *P *< 0.001), and EVLW_a _(*r *= 0.54, *P *= 0.002). A moderate negative correlation was found between P/F ratio and EVLW_p _(*r *= -0.41, *P *= 0.02). Neither lung weight/PBW (*r *= -0.32, *P *= 0.07) nor EVLW_a _(*r *= -0.32, *P *= 0.07) showed a significant correlation with P/F ratio. No correlation was demonstrated between the total pleural effusion amount and EVLW (*r *= 0.006, *P *= 0.97).

### Reference ranges for normal lung weights and calculating normal EVLW_p _values

According to Sawabe and colleagues [38], the normal lung weight values for males and females are 878 ± 339 g (15.1 ± 5.8 g/kg of PBW) and 636 ± 240 g (15.5 ± 5.8 g/kg of PBW), respectively. Table 2 shows calculations of normal EVLW_p _values. In our study, the normal EVLW_p _values were determined to be 7.5 ± 3.3 mL/kg for males and 7.3 ± 3.3 mL/kg for females.

**Table 2:** Calculation of normal extravascular lung water for males and females

Male	Female
EVLW = [0.56 × normal lung weight (in grams)] - 58 = [0.56 × 878] - 58 = 433.7	EVLW = [0.56 × normal lung weight (in grams)] - 58 = [0.56 × 636] - 58 = 298.2
Standard deviation: 189.8	Standard deviation: 134.4
Normal EVLW = 433.7 ± 189.8 mL	Normal EVLW = 298.2 ± 134.4 mL
Normal EVLW_p _= 7.5 ± 3.3 mL/kg	Normal EVLW_p _= 7.3 ± 3.3 mL/kg

EVLW, extravascular lung water; EVLW_p_, extravascular lung water indexed to predicted body weight.

## Discussion

The main findings of this study are that (a) measurement of EVLW using the PiCCO single transpulmonary measurement system is very closely correlated to postmortem lung weight measurement and (b) an EVLW_p _of approximately 7.4 ± 3.3 mL/kg (males 7.5 ± 3.3; females 7.3 ± 3.3) is the reference value for normal lungs.

### Validation and normal value of EVLW

Although a close agreement between EVLW values from PiCCO and gravimetric lung water measurements has been demonstrated in animal models with both direct and indirect lung injury [13-15], there is no conclusive evidence for such agreement in humans. This is the first published report to prove the close correlation of those values in humans with a wide range of illnesses and injured lungs. This correlation was also unaffected by sex, degree of LIS, pleural fluid amount, degree of CI, degree of CVP, degree of PEEP, length of time before the autopsy started, cause of death, or performance of CPR.

Our linear regression equation for the correlation between transpulmonary EVLW measurement and postmortem lung weight (equation 1) is similar to that of Patroniti and colleagues [27] (equation 2), whose EVLW measurements by the thermal-indocyanine green dye double-dilution method showed a good correlation with quantitative computed tomography (CT) findings in 14 mechanically ventilated patients with ARDS. Their equation was as follows:

(2) EVLW (double-indicator)=[0.59×lung weight (CT)]+17.3, where r= 0.7, P<0.01.

We derived statistical values from both the results of the present study and published literature. We calculated linear regression equation 1, which was authenticated statistically with the normal lung weight reference value being substituted in the formula. Data for reference values for normal lung were taken from the study by Sawabe and colleagues [38], which was based on the findings from 1,615 autopsies.

Using this derivation method, we conclude that normal EVLW_p _values for males and females are 7.5 ± 3.3 and 7.3 ± 3.3 mL/kg, respectively. The mean EVLW_p _is approximately 7.4 ± 3.3 mL/kg. These values can be used to distinguish between healthy and pathological lungs.

In our study, EVLW_p _was significantly higher in the RF group (17.1 mL/kg), which consisted of patients with ARDS or pneumonia, than in the non-RF group (10.1 mL/kg), in which most patients had multiple organ failure. The definitive diagnosis was confirmed in autopsy by a pathologist blinded to the study. These values were much higher than the normal EVLW_p _value, 7.4 ± 3.3 mL/kg, especially in the RF group. Several clinical studies have shown increased EVLW_p _documented in patients with ARDS diagnosed by clinical criteria [21,29,33]. To our knowledge, this is the first report showing increased EVLW_p _documented in patients with ARDS or pneumonia confirmed by a pathologist.

### EVLW and pleural effusion

Blomqvist and colleagues [42] found that pleural fluid did not affect the reliability of the double-indicator dilution technique for measuring EVLW in dogs. Deeren and colleagues [43] investigated the effect of thoracentesis on EVLW measurements in eight patients and reported that the fluid in the pleural space did not contribute to the volume traversed by the thermal indicator in single transpulmonary thermodilution measurements in humans. Here, we proved a very close correlation between premortem single transpulmonary thermodilution measurement of EVLW and postmortem lung weight, regardless of the degree of pleural effusion (10 to 1,600 mL).

### Limitations of the study

Despite the statistical significance of the results, the small sample size of this study is its main limitation. Since cardiopulmonary circulation is not a steady-state phenomenon, it is difficult to establish a precise correlation between measurements made premortem and those made postmortem. In addition, CPR was performed in 16 cases (53%) following the final EVLW measurement and this may have affected the postmortem readings. We consider this to be potentially the most serious limitation of our study. However, our data suggest that CPR did not affect the lung weight found at autopsy or the correction between EVLW and lung weight.

Pulmonary inflammation must be taken into consideration, especially among patients with pneumonia. Inflamed cells and purulent matter, including multiple microabscesses, may increase lung weight with or without increasing EVLW values. However, we found no evidence among our study population to support this concern.

EVLW gravimetry, the gold standard of lung water measurement, is a very cumbersome process [44]. In this study, only lung weight was measured. However, measuring a postmortem lung weight is a well-established routine technique that a pathologist performs during an autopsy. Huge volumes of normal and abnormal data of postmortem lung weight have been published and are available. The linear regression equation for a correlation was calculated in order to determine the unknown value, EVLW_p_, from a well-known variable, lung weight. Therefore, we believe that, to gain normal EVLW values, the correlation between EVLW and postmortem lung weight is more significant.

Indicator dilution techniques are also influenced by vascular recruitment and the consequent distribution of zones I and II in the lung because these techniques inherently can detect only perfused lung regions. In addition, it is generally believed that EVLW measured using thermodilution underestimates the true EVLW in the case of heterogeneous lung ventilation/perfusion distribution. We regret that our study design prevented us from demonstrating these issues.

## Conclusions

This human autopsy study has demonstrated that a definite correlation between EVLW measured by the PiCCO system and lung weight in the clinical setting exists independently of illness, sex, degree of lung injury, pleural fluid amount, and degree of CO. We conclude that the normal EVLW_p _value in humans is 7.4 ± 3.3 mL/kg.

## Key messages

• A definite correlation between extravascular lung water, measured by the PiCCO system, and postmortem lung weight in humans exists.

• A normal human value of extravascular lung water indexed by predictive body weight is 7.4 ± 3.3 mL/kg.

## Abbreviations

ARDS: acute respiratory distress syndrome; CI: cardiac index; CO: cardiac output; CPR: cardiopulmonary resuscitation; CT: computed tomography; CV: coefficient of variation; CVP: central venous pressure; EVLW: extravascular lung water; EVLW_A_: extravascular lung water indexed by actual body weight; EVLW_P_: extravascular lung water indexed by predictive body weight; GEDV: global end-diastolic volume; ICC: intraclass correlation coefficient; IQR: interquartile range; ITBV: intrathoracic blood volume; ITTV: intrathoracic thermal volume; LIS: lung injury score; PBW: predicted body weight; PEEP: positive end-expiratory pressure; P/F RATIO: arterial partial pressure of oxygen/fraction of inspired oxygen ratio; PTV: pulmonary thermal volume; RF: respiratory cause of death; SD: standard deviation.

## Competing interests

YY is a member of the Pulsion Medical Systems medical advisory board. The other authors declare that they have no competing interests. There was no financial support for this study.

## Authors' contributions

TT conceived of the study, participated in the design of study, performed the statistical analysis, and helped to draft the manuscript. SK, RT, and TK participated in the study design and helped to draft the manuscript. YY, KM, RO, HH, and HY participated in the study design and provided coordination. TA and TM participated in the design of study. All authors read and approved the final manuscript.
